# The Institutional Worker

**Published:** 1918-05-11

**Authors:** 


THE
### INSTITUTIONAL WORKER
**Being a Special Supplement to "The Hospital"**

### OUR BUREAU OF INFORMATION.

### Rules for Correspondents.

1. Every letter must be accompanied by the coupon to be cut from the back cover (inside page) of The Hospital, current issue, and must contain the name and address of the correspondent with pseudonym for publication if desired. Replies by post cannot be given save under exceptional circumstances at the Editor's discretion.
2. All communications to be addressed to the Editor of The Hospital, 28 Southampton Street, Strand, London, W.C. 2, and marked "Bureau of Information."

### INSTITUTION FACTS AND FIGURES

### A Register of Nurses' Ward Work.

#### Answers to April Question.

The question was:

How should a book be ruled to give at a glance a record of nurses' different duties in male, female, and children's wards, and time spent at classes, etc.? The hospital is a training-school, and nurses take day and night duty. There are three floors for male patients, two for female patients, and a children's block.

#### By "Thorough."

Each hospital has its own hours of duty-time, both on and off, and of course the ward sister arranges each nurse's work. The following scheme will show at a glance where each nurse is situated and her position on the floor in which she is:—

**WARDS.**

| | | Men | | | Women | | Children |
|---|---|---|---|---|---|---|---|
| | | A | B | C | D | E | F |
| Week ending Saturday, April 13, 1918. Nurses on duty | Day | Bell, 1st yr.<br>Jones, 2nd yr.<br>Smith, 3rd yr. | Black, 1st yr.<br>White, 2nd yr.<br>Green, 3rd yr. | Winter, 1st yr.<br>Summer, 2nd yr.<br>Snow, 3rd yr. | Jerkins, 1st yr.<br>Tomkins, 2nd yr.<br>Watkins, 3rd yr. | Todd, 1st yr.<br>Nodd, 2nd yr.<br>Ford, 3rd yr. | Carrol, 1st yr.<br>Farrel, 2nd yr.<br>Darrel, 3rd yr. |
| | Night | Wilson | Brown | Raine | Atkins | Ward | Marrel |

**LECTURES.**

| Date | Ward | Nurse | Day Duty | Off Duty | Time | Lecturer | Subject | Remarks |
|---|---|---|---|---|---|---|---|---|
| | | | A.M. P.M | | P.M. | | | |
| Monday 8/4/18 | A | Bell | 7 to 8.30 | 2 to 4 | 7 to 8 | Dr. Price | Heart | Quick and attentive |
| | | Jones | 7 to 8 30 | 4 to 6 | | | | Thoughtful, but slow |
| | | Smith | 7 to 8.30 | 6 to 10 | | | | Not attentive |
| | B | Black | | | | | | |
| | | White | | | | | | |
| | | | | etc. | etc. | etc. | | |

#### By M. B. M.

I should get a register about 16 in. by 11 in., ruled as below, with an index. It is assumed that the training is for three years, and during that time a complete record of the probationer's work is kept by the authorities. It is also assumed that the class sister, home sister, and ward sister will keep a detailed account of the number of late marks a probationer has, if any, and periodically notify the matron, who will enter them in the register. Also that the class sister will keep a record of the number of classes a probationer attends.

(Male Floors: B, D, E. Female Floors: A, C. Children.)

| Age on Appointment | Name | Previous Occupation |
|---|---|---|
| 26 | | |
| **Date of Appointment** Nov. 12, 1909 | Mary Smith | Governess |

| Particulars | Summary of Reports |
|---|---|
| **Certificate gained** Yes<br>**Conduct** Very good<br>**Work** Satisfactory<br>**Total number of classes attended** ........................<br>**Number of late marks each quarter**<br>1st year ... 1 3 5 —<br>2nd ,, ... — 2 4 —<br>3rd ,, ... 2 3 1 1<br>22=Total<br>**Health** | Mary Smith was a satisfactory probationer throughout her training. She etc. etc. |

| Ward | From | To | Duty | Remarks |
|---|---|---|---|---|
| A. Women's Surgical | Jan. 1 | Feb. 28 | Day | |
| B. Men's Surgical | Feb. 29 | Mar. 30 | ,, | |
| C. Women's Medical | Mar. 31 | April 20 | Night | |
| Children's Surgical | April 21 | May 16 | ,, | |
| D. Men's Medical | May 17 | June 6 | ,, | |
| E. Men's Surgical | June 7 | July 8 | Day | |
| Children's Medical | July 9 | July 30 | | |
| **Holiday** | **July 31** | **Aug. 14** | · | |
| B. Men's Surgical | Aug. 15 | Aug. 25 | ,, | Special for tracheotomy |
| | and so on | and so on | and so on | |

### MAY QUESTIONS.

### (1) The Organisation of a Creche in a Munition Factory.

**A large firm employing about 2,000 women workers, many of whom are married, are anxious to start a creche or day-nursery, for the benefit of the married women, who will be enabled to leave their babies there during working hours. Describe fully the equipment and accommodation required for about fifty babies, and the estimated cost for one year's running. There is already a good welfare department—with two welfare supervisors and two nurses—and it is estimated that the latter could help for a short time each day, and the supervision of the creche would come directly from the head welfare worker.**

### (2) The Protection of Linen in Bedridden Cases.

**In certain Poor-Law infirmaries ointment and oil mixtures are frequently in use for the treatment of backs and pressure points in bedridden cases, especially when wasting diseases are evidenced. What can be recommended as an economical procedure in the matter of personal and bed linen, bearing in mind the effect of grease and oil on linen?**

**The Editor will be glad to receive correspondence and to consider contributions upon all subjects relating to institutional work which affect the welfare of institutional workers.**

### ENQUIRIES AND ANSWERS.

*THE SICK AND IN NEED.*

### Sea-Water Baths.

Both hot and cold sea-water baths are obtainable at the Royal West of England Sanatorium, Weston-super-Mare. No patient can stay longer than a fortnight, as men and women are taken alternately. The terms of admission are 31s. per week. Apply to the Hon. Lady Superintendent for further particulars.—Deirdre.

### Defective Speech.

The question of a remedy depends upon the cause of your defective speech—that is, whether it be due to habit and imperfect training or to physical defect. We suggest that you consult your doctor in this matter.—D. C. L.

### Rest Home for Convalescent V.A.D. Nurse.

We suggest that you apply to the Devonshire House Welfare Department for admission to one of their Homes for the purpose, such as Ardington House, in the north-west of Berkshire, kindly lent by Lady Wantage. There is also a smaller Home, near Warwick, which accommodates six nursing members employed in military hospitals. The first-named home has accommodation for twenty patients.—Sibyl.

### Help for Disabled Nurse.

The Trained Nurses' Annuity Fund for Disabled Nurses, 73 Cheapside, E.C., may possibly help the nurse in whom you are interested. This Fund grants annuities to disabled trained nurses of at least ten years' service who are not less than forty years of age. We suggest that the nurse should write to the Hon. Secretary at the address given above. A coupon should be sent with each inquiry. We regret we cannot give postal replies.—E. S.

*MISCELLANEOUS.*

### The Training of Dental Mechanics.

We do not at present know of any school for the training of dental mechanics. A well-known private institution which provided instruction of this character is now closed. The usual method of training is by apprenticeship to a dental surgeon. There are several firms which do mechanical work for the profession, and it is possible that these receive pupils, as, for instance, Claudius Ash and Co., Broad Street, Golden Square, W.; The Dental Manufacturing Co., Alston House, Newman Street, Oxford Street, W. You might also apply to the Dental Department at Guy's Hospital.

